# Prevalence of sensitization to molecular food allergens in Europe: A systematic review

**DOI:** 10.1002/clt2.12175

**Published:** 2022-07-06

**Authors:** Daniil Lisik, Athina Ioannidou, Giulia Spolidoro, Mohamed Ali, Sungkutu Nyassi, Yohanes Amera, Graciela Rovner, Ekaterina Khaleva, Carina Venter, Ronald van Ree, Margitta Worm, Berber Vlieg‐Boerstra, Aziz Sheikh, Antonella Muraro, Graham Roberts, Bright I. Nwaru

**Affiliations:** ^1^ Krefting Research Centre University of Gothenburg Gothenburg Sweden; ^2^ Department of Clinical Science and Community Health University of Milan Milan Italy; ^3^ Division of Physiotherapy Department of Neurobiology, Care Sciences and Society Karolinska Institutet Stockholm Sweden; ^4^ ACT Institutet Sweden Gothenburg Sweden; ^5^ Faculty of Medicine University of Southampton Southampton UK; ^6^ Section of Allergy & Immunology University of Colorado Denver School of Medicine and Children's Hospital Denver Colorado USA; ^7^ Department of Experimental Immunology and Department of Otorhinolaryngology Academic Medical Center Amsterdam The Netherlands; ^8^ Division of Allergy and Immunology Department of Dermatology, Allergy and Venerology Charité Universitätsmedizin Berlin Berlin Germany; ^9^ Department of Pediatrics OLVG Hospital Amsterdam The Netherlands; ^10^ Usher Institute University of Edinburgh Edinburgh UK; ^11^ Department of Mother and Child Health University of Padua Padua Italy; ^12^ David Hide Asthma and Allergy Research Centre St Mary's Hospital Newport UK; ^13^ Wallenberg Centre for Molecular and Translational Medicine University of Gothenburg Gothenburg Sweden

**Keywords:** epidemiology, food allergy, molecular allergen, sensitization, systematic review

## Abstract

**Background:**

Recent reports indicate that the prevalence of food allergy is increasing, but accurate estimates remain a challenge due to cross‐reactivity and limited use of precise diagnostic methods. Molecular allergy diagnostics, in which sensitization to individual molecular allergens is measured, is emerging as a promising tool for evaluation of sensitization profiles. In this systematic review, we summarized estimates of prevalence of sensitization to molecular food allergens in the general population in Europe.

**Methods:**

Following a protocol prospectively registered with the International Prospective Register of Systematic Reviews (PROSPERO; reference CRD42021266657), we searched seven databases with no restrictions on publication date or language. Two reviewers independently screened the literature, extracted data, and appraised the risk of bias in the included studies. The findings were synthesized narratively.

**Results:**

From 4776 de‐duplicated records, five studies, with low to moderate overall risk of bias, were included. Forty‐six molecular allergens from 18 foods were investigated. Overall, the prevalence of sensitization was low, particularly for major allergens, and non‐existent for 10 molecular allergens (0% [95% CI 0–0.8]). The highest prevalence was seen for PR‐10 proteins, such as Cor a 1.04 (13.6% [95% CI 10.9–16.9]).

**Conclusions:**

Available data, primarily from North‐western Europe, indicate that sensitization to molecular food allergens is overall low. The highest estimates were found for cross‐reactive PR‐10 proteins. There were not enough studies to discern regional differences or perform meta‐analysis, highlighting the need for more population‐representative studies in order to elucidate patterns of sensitization to molecular food allergens in Europe.

## 1 INTRODUCTION

Food allergy has become increasingly common in recent years, both in economically‐developed and ‐developing countries, constituting a significant public health burden.^1^, ^2^, ^3^ In a previous systematic review published in 2014 (based on papers from 2000 to 2012) for the European Academy of Allergy and Clinical Immunology (EAACI),^4^ we estimated the point prevalence of self‐reported food allergy to any food in Europe to be 5.9% (95% confidence interval [CI] 5.7–6.1). Assessed with the “gold standard”^5^ method of double‐blind placebo‐controlled food challenge (DBPCFC), the estimate was 0.9% (95% CI 0.8–1.1). As DBPCFC is resource‐intensive and time‐consuming, this method is seldomly used in epidemiologic studies or outside of the academic context.^4^, ^5^, ^6^, ^7^

At the molecular level, foods such as peanut are composed of multiple components, some of which are classified as molecular allergens.^8^ Similarity in structure and properties of molecular allergens can give rise to cross‐reactivity between different foods, as well as between foods and other types of allergen sources, for example, inhalant allergen sources such as pollen and mites.^9^ Cross‐reactivity is commonly expressed with mild symptoms of the oral mucosa following ingestion of certain foods, but may also present with severe systemic reactions, depending on the cross‐reactive allergen, such as lipid transfer proteins (LTPs).^10^ Traditionally, in vitro diagnostics of food allergy has involved measurement of specific immunoglobulin E (sIgE) sensitization to whole allergen extracts. In contrast, molecular allergy diagnostics has enabled measurement of sensitization to individual molecular allergens.^11^, ^12^ Molecular allergy diagnostics is a promising tool to establish the source of primary sensitization and the origins and patterns of cross‐reactivity.^11^, ^13^, ^14^, ^15^, ^16^ The advent of biochip technology, where sensitization to an array of molecular allergens can be tested simultaneously using small quantities of serum,^17^, ^18^ has further increased the utility of molecular allergy diagnostics, particularly in complicated cases^19^, ^20^ and to produce rich sensitization profiles.^12^, ^20^

As management of food allergy includes avoidance of foods, and in some cases resource‐intensive immunotherapy,^21^, ^22^, ^23^ it is important to establish correct diagnosis. In the last 2 decades, a large body of research on molecular allergy diagnostics has been produced.^12^, ^24^ However, to the best of our knowledge, no systematic review has been undertaken to synthesize the existing literature and elucidate the sensitization patterns to molecular food allergens across Europe in the general population. Therefore, in updating the previous EAACI‐commissioned systematic review,^4^ our aim in this systematic review was to estimate the prevalence of sensitization to molecular food allergens in the general population in Europe as defined by sensitization to molecular food allergens.

## 2 METHODS

### 2.1 Protocol

This systematic review was performed according to a protocol, which was reported according to the Preferred Reporting Items for Systematic Review and Meta‐Analysis Protocols (PRISMA‐P).^25^ The protocol was prospectively registered in the International Prospective Register of Systematic Reviews (PROSPERO; https://www.crd.york.ac.uk/prospero/display_record.php?RecordID=266657; reference CRD42021266657).

### 2.2 Study eligibility criteria

#### 2.2.1 Study design, language, and publication status

Systematic reviews and meta‐analyses, prospective and retrospective cohort studies, cross‐sectional studies, and case‐control studies were eligible for inclusion. Conversely, case studies, case series, clinical trials, non‐systematic reviews, expert opinions, and animal studies were excluded. There was no restriction regarding publication status/date or language. Articles in languages other than English were translated using Google Translate.^26^

#### 2.2.2 Participants

Studies with population‐representative samples were eligible. A sample was considered population‐representative if the participants had comparable statistical likelihood of being recruited, either through the means of exhaustive sampling, or through random selection. Studies with participants residing in European countries, as defined by the United Nations (with addition of countries geographically proximate to Europe; see Supporting Information S1),^27^ were included. There were no restrictions on sample size, gender, age, or medical background.

#### 2.2.3 Outcomes

Sensitization to any molecular food allergen, as assessed by sIgE above a study‐defined threshold, using any molecular allergy diagnostic method. Studies which did not present data for each individual or the percentage of sensitized individuals were excluded.

### 2.3 Search methods

Allergome, CINAHL, the Cochrane Library, EMBASE, MEDLINE, Scopus, and Web of Science were searched. These databases were selected to keep in line with previous systematic reviews on the topic as well as what has been previously suggested to attain a comprehensive coverage of relevant journals.^4^, ^28^ The databases were searched for articles published from inception up until June 30, 2021 (except Allergome, which was searched on September 1, 2021). The search queries were structured as four blocks concatenated with the “AND” Boolean operator. The search blocks contained, respectively, keywords for: 1) food allergy, foods, and molecular allergens; 2) epidemiological measurements (e.g., “prevalence”); 3) molecular allergy diagnostic‐related terms (e.g., “molecular diagnos*”); 4) Europe and its countries. The search strategy was revised through scoping searches on PubMed, in which additional keywords were identified. The Peer Review of Electronic Search Strategies (PRESS)^29^ guidelines were used to revise the search strategy. Reference lists of included articles were reviewed for relevant studies.

Allergome was searched by: 1) searching for the first molecular allergen (in alphanumeric order) of that food (e.g., “Ara h 1” for peanut); 2) browsing to the page for each molecular allergen of that food (e.g., https://www.allergome.org/script/dettaglio.php?id_molecule=50 for Ara h 1); 3) fetching articles in the “Epidemiology from Literature” table. Due to the overwhelming number of allergens in Allergome, steps 1)–3) were repeated only for the eight most common foods.^30^, ^31^ The search queries are detailed in Supporting Information S2.

### 2.4 Data management

EndNote 20 (Clarivate Analytics, 2020) was used for de‐duplication. Screening of title/abstract, full‐text screening, and general management of full‐text PDFs was done in Rayyan (https://rayyan.ai).

### 2.5 Screening and selection

The first stage of screening was based on title/abstract. Irrelevant articles, and those meeting any exclusion criterion, were excluded. In the second stage, the full‐text of each article was screened. This process was performed by two reviewers (DL and AI) independently. After each stage, the decisions were unblinded and compared for differences, which were resolved through discussion. No arbitration through a third reviewer was required.

### 2.6 Data extraction

A Microsoft Excel (Microsoft Corporation, 2021) extraction form (available upon request), piloted a priori, was used to extract data. Two reviewers (DL and AI) extracted data independently, and differences were resolved through discussion. No arbitration through a third reviewer was required.

### 2.7 Data items

The following data items were extracted and summarized from each study: surname of first author; publication year; study design; number of individuals recruited/included; period of data collection; country of study; subject characteristics (age, gender, and epidemiological/medical background); molecular allergen(s) investigated; diagnostic method and cut‐off value(s); results (e.g. sensitization prevalence).

### 2.8 Assessment of risk of bias

Assessment of risk of bias in the included studies was done using forms based on the Critical Appraisal Skills Programme (CASP) tool. CASP checklists for systematic reviews, cohort studies (reused for cross‐sectional studies), and case‐control studies, respectively, were adapted. Specific questions differed somewhat between the forms for respective study design, but assessed the same four sections: study design, participant selection, exposure assessment, and outcome assessment. Each of these sections were rated as “high”, “moderate”, or “low” risk of bias, or “not applicable”/“not available”, depending on available information. Each study was also given an overall rating, based on the section ratings and a collected assessment. This process was performed by two reviewers (DL and AI) independently. Differences were resolved through discussion. No arbitration through a third reviewer was required.

### 2.9 Data synthesis

A descriptive table was produced to summarize essential characteristics of the included studies. The results were narratively synthesized. For consistency, data in the synthesis were limited to measurements with the conventional^32^, ^33^, ^34^ cut‐off values of 0.35 kUA/l for ImmunoCAP and 0.3 ISU for ISAC. Meta‐analysis was not possible to perform, given the insufficient and disparate data. The Preferred Reporting Items for Systematic Reviews and Meta‐Analyses (PRISMA)^35^ checklist (Supporting Information S3) was used to guide the reporting of this systematic review.

### 2.10 Statistical analyses

Prevalence was defined as $p=n_{\text{sensitized}}/n_{\text{total}}$. The 95% CI was estimated using Wilson's score interval without continuity correction,^36^, ^37^ with $p_{\text{lower}}=\frac{2r\;+\;z^{2}\;-\;z\sqrt{z^{2}\;+\;4rq}\;}{2\left(n\;+\;z^{2}\right)}$ and $p_{\text{upper}}=\frac{2r\;+\;z^{2}\;+\;z\sqrt{z^{2}\;+\;4rq}\;}{2\left(n\;+\;z^{2}\right)}$, where *r *=* n*_sensitized_, *q *= 1 –* p*, and *z *= 1.96 (the 97.5th percentile value from the standard normal distribution for *α *= 0.05).

### 2.11 Deviations from the protocol

After having conducted the initial searches, the authors identified Allergome as a relevant source, housing articles not identified with previous methods. A search of Allergome was thus performed *post hoc*.

## 3 RESULTS

### 3.1 Study selection and characteristics

**FIGURE 1:**
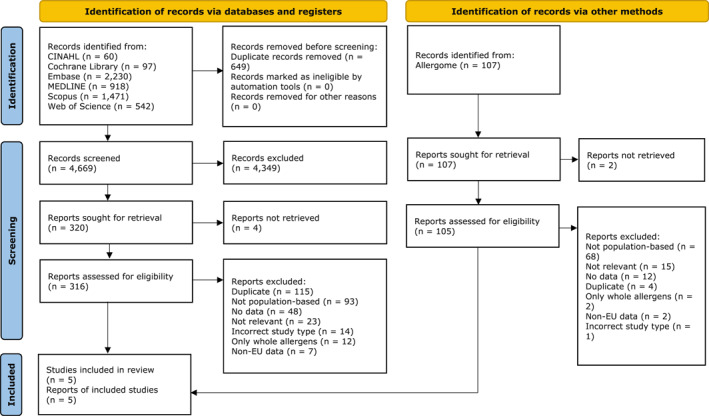
PRISMA flow diagram of the literature search and selection of records

**FIGURE 2:**
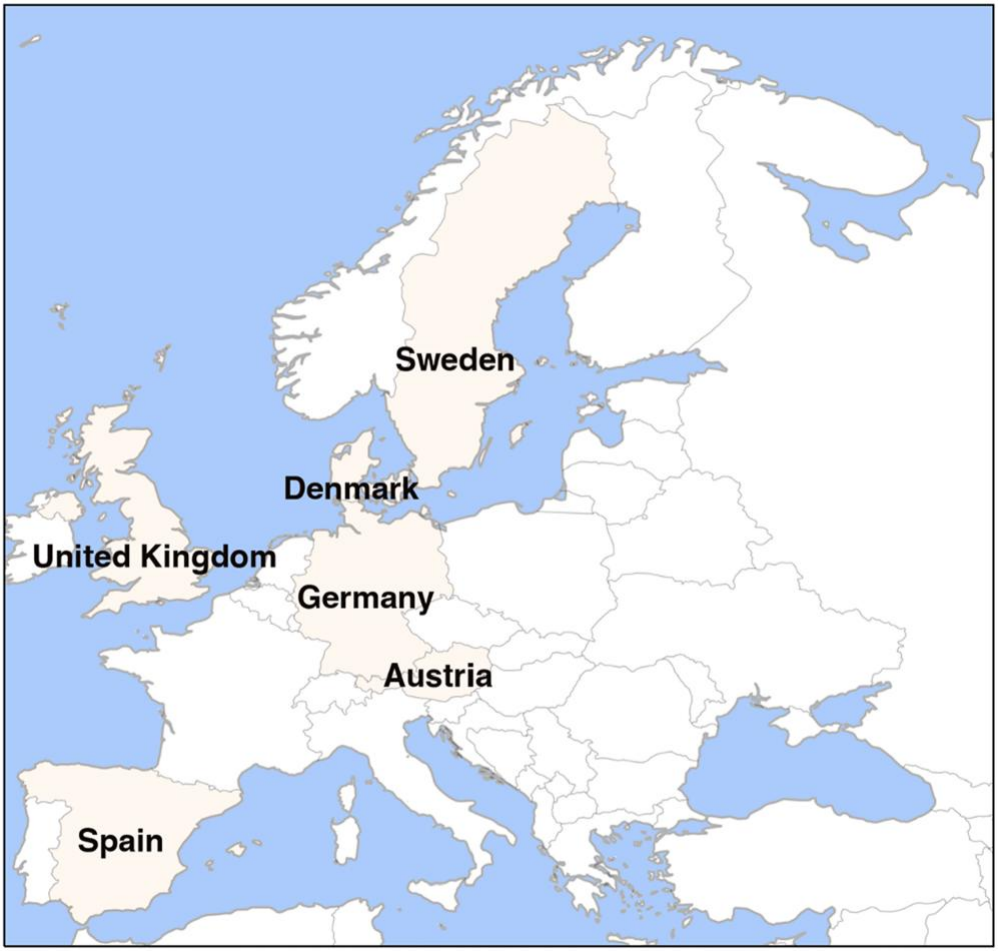
Map of Europe, highlighting the countries where the included studies took place

Our searches yielded 5425 records. After de‐duplication, 4776 records were screened. Of these, five studies^38^, ^39^, ^40^, ^41^, ^42^ were included in the systematic review. Figure 1 illustrates the PRISMA flow diagram of this process. Across the five studies, 46 molecular allergens from 18 foods were investigated, of which peanut and wheat were the most frequently examined. One study^41^ presented period prevalence estimates and time trends data, while the remaining data estimated point prevalence. There were two cross‐sectional studies^40^, ^42^ and two cohort studies.^39^, ^41^ The fifth study^38^ was of nested case‐control study design. Participants were either part of population‐based cohorts^38^, ^39^, ^41^ or were comprehensively recruited from the region.^40^, ^42^ Four studies^38^, ^39^, ^41^, ^42^ were conducted in North‐western Europe, with subjects aged 8–21 years. The fifth study^40^ recruited adults from Northern and Southern Europe, respectively (Figure 2). The smallest study examined 104 participants,^41^ while the largest sample consisted of 2741 individuals.^40^ Three studies used ImmunoCAP,^38^, ^39^, ^40^ with one^40^ using both the conventional^32^ cut‐off of 0.35 kUA/l and the lower cut‐off of 0.1 kUA/l, while the two other studies^38^, ^39^ only used 0.35 kUA/l. The two remaining studies^41^, ^42^ used ISAC, and the conventional^32^ cut‐off of 0.3 ISU. Further details of the included studies are found in Table 1.

**TABLE 1:** Characteristics of included studies

Study *(first author and year)*	Study design	Subjects participating *(n [% of recruited])*	Period of data collection	Country of study	Subject characteristics *(1) age; 2) n men (%); 3) epidemiological/medical background)*	Sensitization prevalence *(% [cases/total; 95% CI])*	Asssessment *(method, cut‐off)*
Asarnoj^38^ 2010	Nested case‐control study	200 (N/A)	2002–2004 (approx.)	Sweden	1) ∼8 (8 years follow‐up); 2) N/A (not significantly different from the baseline cohort); 3) BAMSE birth cohort	***Point prevalence***	ImmunoCAP, ≥0.35 kUA/l
						**Ara h 1**	
						*All*: 14.5 (29/200; 10.3–20.1); *peanut‐sensitized (tolerance unknown)*: 50 (25/50; 36.6–63.4); *peanut and birch pollen‐sensitized*: 8 (4/50; 3.2–18.8); *birch pollen‐sensitized*: 0 (0/50; 0–7.1); *not sensitized to nor birch nor peanut*: 0 (0/50; 0–7.1); *peanut allergy (history)*: 45.2 (28/62; 33.4–57.5); *peanut‐tolerant (history)*: 0.7 (1/136; 0.1–4.1)	
						**Ara h 2**	
						*All*: 26.5 (53/200; 20.9–33); *peanut‐sensitized (tolerance unknown)*: 70 (35/50; 56.3–80.9); *peanut and birch pollen‐sensitized*: 36 (18/50; 24.1–49.9); *birch pollen‐sensitized*: 0 (0/50; 0–7.1); *not sensitized to nor birch nor peanut*: 0 (0/50; 0–7.1); *peanut allergy (history)*: 72.6 (45/62; 60.4–82.1); *peanut‐tolerant (history)*: 5.2 (7/136; 2.5–10.2)	
						**Ara h 3**	
						*All*: 12.5 (25/200; 8.6–17.8); *peanut‐sensitized (tolerance unknown)*: 42 (21/50; 29.4–55.8); *peanut and birch pollen‐sensitized*: 8 (4/50; 3.2–18.8); *birch pollen‐sensitized*: 0 (0/50; 0–7.1); *not sensitized to nor birch nor peanut*: 0 (0/50; 0–7.14); *peanut allergy (history)*: 38.7 (24/62; 27.6–51.2); *peanut‐tolerant (history)*: 0.7 (1/136; 0.1–4.1)	
						**Ara h 8**	
						*All*: 15 (30/200; 10.7–20.6); peanut‐sensitized (tolerance unknown): 0 (0/50; 0–7.1); *peanut and birch pollen‐sensitized:* 38 (19/50; 25.9–51.9); *birch pollen‐sensitized*: 22 (11/50; 12.8–35.2); *not sensitized to nor birch nor peanut*: 0 (0/50; 0–7.1); *peanut allergy (history)*: 14.5 (9/62; 7.8–25.3); *peanut‐tolerant (history)*: 15.4 (21/136; 10.3–22.5)	
Gonzalez‐Quintela^40^ 2014	Cross‐sectional study	2741 (66.4)	2011–2012 (Denmark), 2000–2001 (Spain)	Denmark, Spain	*Denmark cohort (pre‐exclusion)*: 1) median 55.7 years (range 24–76); 2) 1057 (45.8); 3) health 2006 cohort; *Spain cohort (pre‐exclusion)*: 1) 54 (18–92); 2) 206 (43.9); 3) A‐Estrada survey	***Point prevalence***	ImmunoCAP, ≥0.1/0.35 kUA/l
						**Alpha‐gal**	
						*Denmark cohort (≥0.1 kUA/l)*: 5.6 (128/2297; 4.7–6.6); *Denmark cohort (≥0.35 kUA/l)*: 1.8 (42/2297; 1.4–2.5); *Spain cohort (≥0.1 kUA/l)*: 8.1 (36/444; 5.9–11); *Spain cohort (≥0.35 kUA/l):* 2.3 (10/444; 1.2–4.1)	
Huang^41^ 2018	Cohort study	104 (7.9)	2000 (approx.)	Germany	1) ∼10 (10 years follow‐up); 2) 55 (52.9); 3) German Multicentre Allergy Study (MAS) birth cohort	***Period prevalence***	ISAC, ≥0.3 ISU
						**Act d 2**	
						*All (age 1–10 years)*: 7.7 (8/104; 3.9–14.5)	
						***Time‐trends (1–10 years)***	
						**Act d 1, Act d 5, Ana o 2, Ara h 1, Ara h 2, Bos d 4, Bos d 5, Bos d 8, Bos d lactoferrin, Cor a 9, Gal d 3, Ses i 1**	
						Barely detectable throughout first decade	
						**Act d 2**	
						Frequently detectable and increasing throughout first decade	
						**Ara h 3, Gly m 5, Gly m 6, Tri a 19, Tri a 30**	
						Never detectable	
						**Gal d 1, Gal d 2**	
						Higher prevalence during preschool versus school age	
Stemeseder^42^ 2017	Cross‐sectional study	501 (76)	2013–2014	Austria	1) 15.2 (range 12–21); 2) 222 (44.3); 3) pupils in Salzburg attending grade 8–13	***Point prevalence (ordered in increasing prevalence)***	ISAC, ≥0.3 ISU
						**Act d 5, Ara h 1–3, Ara h 6, Ara h 9, Ber e 1, Bos d 4–6, Bos d 8, Bos d lactoferrin, Cor a 8, Cor a 9, Fag e 2, Gal d 1, Gly m 5, Gly m 6, Jug r 1, Jug r 3, Pru p 3, Ses i 1, Tri a 14, Tri a 19.0101, Tri a 30**	
						*All*: 0 (0/501; 0–0.8)	
						**Act d 1, Gad c 1, Gal d 2, Pen m 4**	
						*All*: 0.2 (1/501; 0–1.1)	
						**Pen m 1**	
						*All*: 0.4 (2/501; 0.1–1.4)	
						**Ana o 2, Gal d 3, Gal d 5**	
						*All*: 0.6 (3/501; 0.2–1.8)	
						**Act d 2, Act d 8, Pen m 2**	
						*All*: 2 (10/501; 1.1–3.6)	
						**Api g 1**	
						*All*: 2.8 (14/501; 1.7–4.6)	
						**Gly m 4**	
						*All*: 5.9 (30/501; 4.2–8.4)	
						**Ara h 8**	
						*All*: 7.9 (40/501; 5.9–10.7)	
						**Jug r 2**	
						*All*: 8.4 (42/501; 6.3–11.1)	
						**Pru p 1**	
						*All*: 10.6 (53/501; 8.2–13.6)	
						**Mal d 1**	
						*All*: 11.8 (59/501; 9.2–14.9)	
						**Cor a 1.04**	
						*All*: 13.6 (68/501; 10.9–16.9)	
Venter^39^ 2016	Cohort study	827 (N/A)	2011–2012 (approx.)	United Kingdom	1) ∼10 (10 years follow‐up); 2) N/A; 3) Food Allergy and Intolerance Research (FAIR) birth cohort	***Point prevalence***	ImmunoCAP, ≥0.35 kUA/l
						**Tri a 14**	
						*Wheat‐sensitized*: 2.7 (1/37; 0.5–13.8)	
						**Tri a 19**	
						*Wheat‐sensitized*: 8.1 (3/37; 2.8–21.3)	
						**Wheat gliadin**	
						*Wheat‐sensitized*: 2.7 (1/37; 0.5–13.8)	

*Note*: Bold value indicates individual molecular allergens for which there are sensitization data.

Abbreviations: 95% CI, 95% confidence interval; IQR, interquartile range; ISAC, Immuno Solid‐phase Allergy chip; ISU, ISAC Standardized Units; kUA/l, Kilo‐units of allergen‐specific immunoglobulin E levels.

### 3.2 Assessment of risk of bias

Two studies^40^, ^41^ were rated as having an overall “low” risk of bias, while three studies^38^, ^39^, ^42^ were rated as “moderate” (Table 2).

**TABLE 2:** Assessment of risk of bias in the included studies

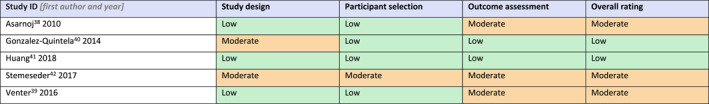

### 3.3 Point prevalence

#### 3.3.1 Alpha‐gal

Alpha‐gal was investigated by Gonzalez‐Quintela et al.,^40^ who randomly recruited 2741 individuals from the general population, with an estimated sensitization prevalence in Denmark of 1.8% (95% CI 1.4–2.5), and 2.3% (95% CI 1.2–4.1) in Spain. None of the subjects sensitized to alpha‐gal in this study population had alpha‐gal syndrome.

#### 3.3.2 Peanut

**FIGURE 3:**
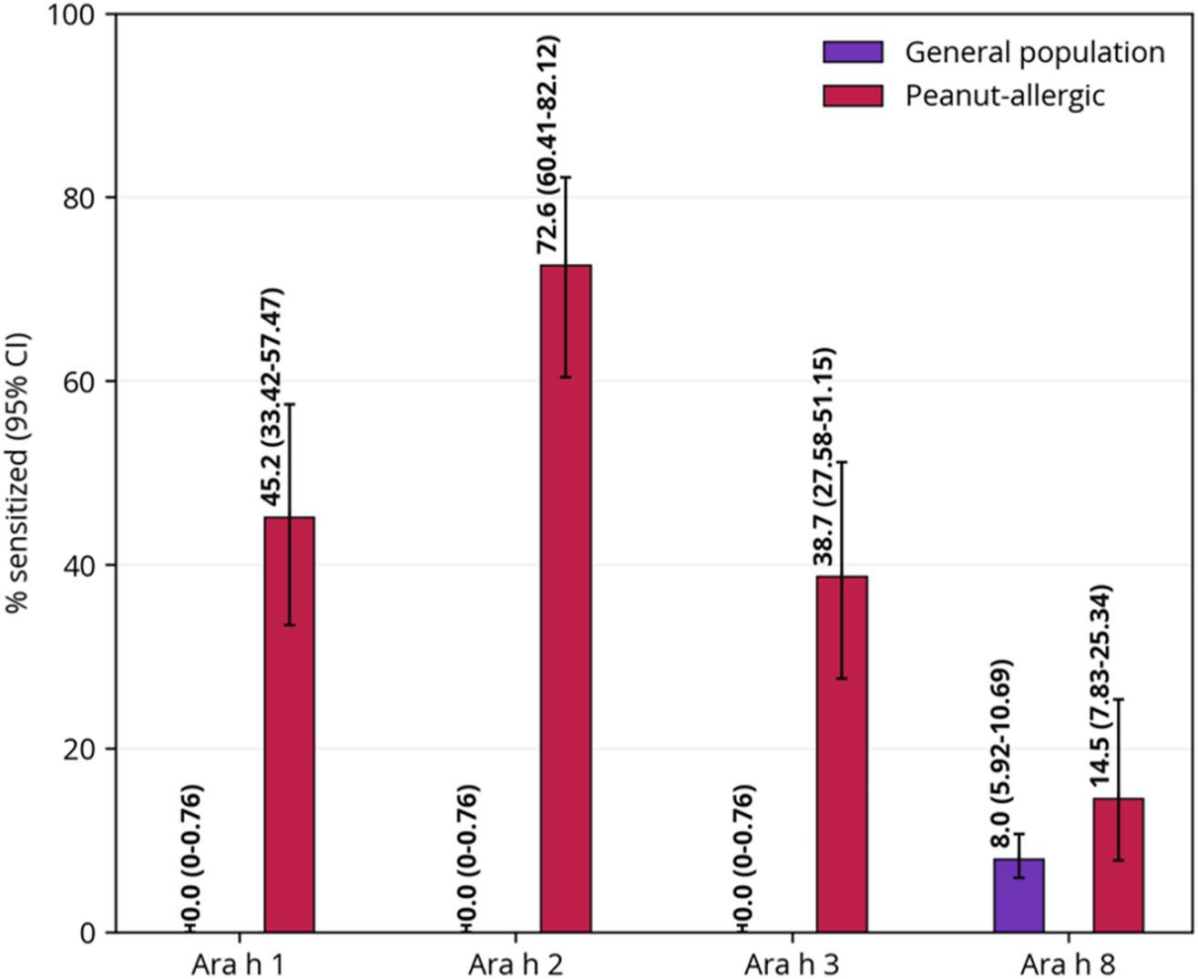
Point prevalence of sensitization to molecular peanut allergens investigated in two or more studies. Data from the red bars (“Peanut‐allergic”) are from Asarnoj et al.,^37^ and were estimated using ImmunoCAP™ (cut‐off ≥ 0.35 kUA/l). Data from the “General population” are from Stemeseder et al.,^41^ using ImmunoCAP™ ISAC (cut‐off ≥ 0.3 ISAC specific units [ISU]). The participants from Stemeseder et al. were slightly older (12–21 years) than the participants from Asarnoj et al. (∼8 years). ISAC, Immuno Solid‐phase Allergy chip

Molecular peanut allergens were evaluated in two studies.^38^, ^42^ Differences in sensitization prevalence are illustrated in Figure 3. Asarnoj et al.^38^ performed a nested case‐control study within a Swedish birth cohort at the time of the eight‐year follow‐up, assessing sensitization to Ara h 1–3 and Ara h 8. Four groups, each consisting of 50 children, representing four different sensitization patterns to whole peanut and birch pollen, were randomly sampled. They found point prevalence estimates of ≥42% to each of the major peanut allergens Ara h 1–3 in those sensitized to peanut but not birch pollen, apart from Ara h 8, to which none in this group were sensitized. In contrast, in the group sensitized to both peanut and birch pollen, sensitization was most common to Ara h 8 at 38% (95% CI 25.9–51.9), 8% (95% CI 3.2–18.8) to Ara h 1 and 3, respectively, and 36% (95% CI 24.1–49.9) to Ara h 2. In those not sensitized to neither birch pollen nor peanut, none were sensitized to any of the four molecular peanut allergens. In those who reported no history of peanut allergy, sensitization was seen in 0.7% (95% CI 0.1–4.1) to Ara h 1 and Ara h 3, respectively, 5.2% (95% CI 2.5–10.2) to Ara h 2, and 15.4% (95% CI 10.3–22.4) to Ara h 8. In the group who reported allergic symptoms to peanut, sensitization was seen for Ara h 1 in 45.2% (95% CI 33.4–57.5), Ara h 2 in 72.6% (95% CI 60.4–82.1), Ara h 3 in 38.7% (95% CI 27.6–51.2), and Ara h 8 in 14.5% (95% CI 7.8–25.3).

In a sample of 501 school‐age children in Austria, Stemeseder et al.^42^ found no sensitization to Ara h 1–3, Ara h 6, or Ara h 9 (each 0% [95% CI 0–0.8]). The point prevalence of sensitization to Ara h 8 was 7.9% (95% CI 5.9–10.7).

#### 3.3.3 Wheat

**FIGURE 4:**
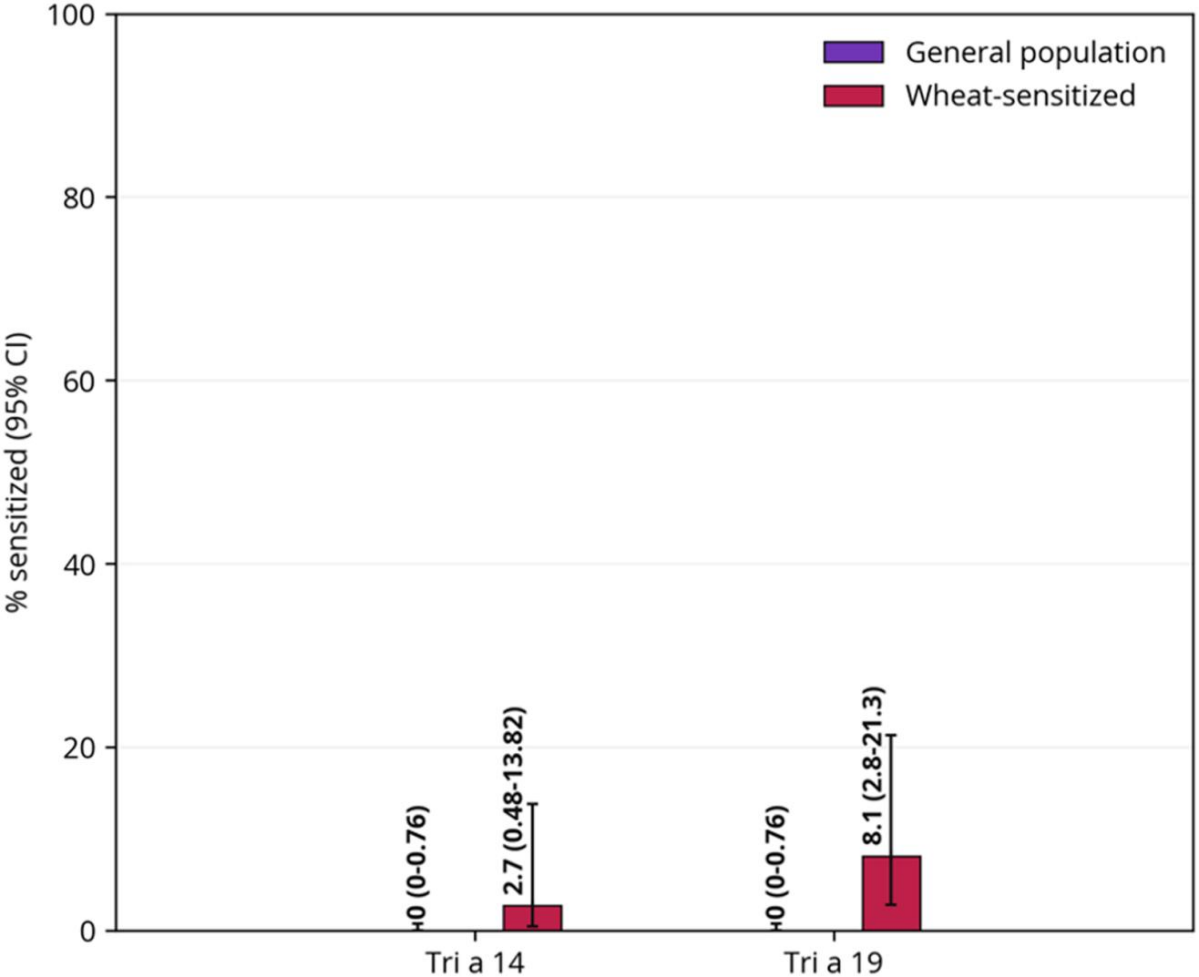
Point prevalence of sensitization to molecular wheat allergens investigated in two or more studies. Data from the red bars (“Wheat‐sensitized”) are from Venter et al.,^38^ and were estimated using ImmunoCAP™ (cut‐off ≥ 0.35 kUA/l). Data from “General population” are from Stemeseder et al.,^41^ using ImmunoCAP™ ISAC (cut‐off ≥ 0.3 ISAC specific units [ISU]). Participants from the two aforementioned studies were of similar age (8–10 years). ISAC, Immuno Solid‐phase Allergy chip

Molecular wheat allergens were evaluated in two studies.^39^, ^42^ Figure 4 illustrates sensitization to molecular allergens investigated in two studies. In the study by Venter et al.,^39^ a subset (*n* = 246) of children from a British birth cohort followed up at 10 years (*n* = 827) consented to an allergy screening blood test using a panel of aeroallergens and food allergens, respectively. In the 15% (*n* = 37) who showed sIgE sensitization to whole wheat extract, molecular allergy diagnostics was performed. Prevalence of sensitization to Tri a 14 and wheat gliadin (mix of *α, β*, *γ*, and *ω* gliadins), respectively, was 2.7% (95% CI 0.5–13.8), and 8.1% (95% CI 2.8–21.3) to Tri a 19 in the wheat‐sensitized children. The authors found that significantly more subjects (*p* < 0.05) had a history of any allergen sensitization among those who took the allergy screening test than those who did not take the test, and thus regard that the prevalence estimates are reflective of a higher risk population. Stemeseder et al.,^42^ investigating sensitization profiles in 501 school‐age pupils in Austria, found no sensitization (0% [95% CI 0–0.8]) to Tri a 14, Tri a 19, or Tri a 30.

#### 3.3.4 Other allergens

In the study by Stemeseder et al.,^42^ 501 school‐age children in Austria were assessed for sensitization to an array of molecular allergens. For 17 molecular allergens (Act d 5, Ber e 1, Bos d 4‐6, Bos d 8, Bos d lactoferrin, Cor a 8, Cor a 9, Fag e 2, Gal d 1, Gly m 5, Gly m 6, Jug r 1, Jug r 3, Pru p 3, and Ses i 1), sensitization was non‐existent (0% [95% CI 0–0.8]). Furthermore, 14 molecular allergens had sensitization estimates <10% (Act d 1, Act d 2, Act d 8, Gad c 1, Gal d 2, Pen m 4, Pen m 1, Ana o 2, Gal d 3, Gal d 5, Pen m 2, Api g 1, Gly m 4, and Jug r 2). For the PR‐10 proteins Pru p 1, Mal d 1, and Cor a 1.04, estimates were slightly higher: 10.6% (95% CI 8.2–13.6) for Pru p 1, 11.8% (95% CI 9.2–14.9) for Mal d 1, and 13.6% (95% CI 10.9–16.9) for Cor a 1.04.

### 3.4 Period prevalence

In a birth cohort of 104 children from five German cities, Huang et al.^41^ estimated the period prevalence of sensitization to Act d 2 during the age of 1–10 years to 7.7% (95% CI 3.9–14.5).

### 3.5 Time trends

Huang et al.^41^ found, in their German birth cohort population of 104 subjects, that sIgE to 17 molecular allergens (Act d 1, Act d 5, Ana o 2, Ara h 1–3, Bos d 4, Bos d 5, Bos d 8, Bos d lactoferrin, Cor a 9, Gal d 3, Gly m 5, Gly m 6, Ses i 1, Tri a 19, and Tri a 30) was barely/never detectable throughout the first decade of life. Sensitization prevalence to Gal d 1–2 was higher at preschool age than in school age, and increasing for Act d 2 throughout this period.

## 4 DISCUSSION

### 4.1 Summary of principal findings

This is the first systematic review to summarize the sensitization prevalence to molecular food allergens in the general population in Europe. Overall, sensitization was low to most of the 46 investigated molecular allergens. In non‐allergic individuals, labile, cross‐reactive molecular allergens were the most common, while individuals with food allergy were more commonly sensitized to storage proteins. Sensitization to 20 molecular allergens was non‐existent or barely detected. In the general population, the highest sensitization was found for PR‐10 proteins. There were too few studies to draw conclusions regarding which foods had the highest/lowest sensitization rates, and whether the “big eight” foods are the most common at the molecular allergen level. Some indications of regional differences were noted, such as lower sensitization to alpha‐gal in Denmark than in Spain, but the data were insufficient to draw any conclusions, neither on country level nor on urban/rural level. It is also noteworthy, that while the included studies were published 2010–2018, the data were collected 2000–2014; thus, a significant proportion of the data describes sensitization prevalence from the early 21^st^ century.

### 4.2 Strengths and limitations

We have conducted a comprehensive search of seven databases with no restrictions on language or publication date. Despite this, the number of relevant studies with population‐based samples was low. Given the vast nomenclature in molecular allergology, the search queries may not have encompassed all appropriate keywords, and thus not have identified all relevant literature. The included studies were heterogenous, which limited the possibility to identify patterns from the findings or perform meta‐analysis. Such differences were apparent in the molecular allergy diagnostic assays employed across studies. In addition, four out of the five studies only presented data on children/adolescents, and four studies only presented data from North‐western Europe, further narrowing the representativeness of the data. It is also worth noting, that two of the studies employed ISAC, which, due to lower sensitivity, increases the risk of underestimating the point prevalence compared to ImmunoCAP.^34^ Finally, results may have been affected by the composition of the test material, particularly from varying amounts of cross‐reactive carbohydrate determinants (CCDs).^8^ Even in recombinant allergens, false positive results may have occurred in individuals with high anti‐CCD IgE levels, due to CCDs in solid‐phase allergen carriers of cellulose.^43^

### 4.3 Comparison to previous and related literature

To the best of our knowledge, no systematic review has previously assessed the prevalence of sensitization to molecular allergens of multiple foods on a Europe‐wide general population basis. Existing reviews have primarily focused on single foods in a non‐systematic manner,^44^, ^45^ or on clinical aspects, such as sensitivity and specificity of molecular allergy diagnostic methods for certain foods.^46^, ^47^, ^48^ In terms of primary research, data from the majority of related studies^49^, ^50^, ^51^, ^52^, ^53^, ^54^, ^55^, ^56^, ^57^ are from specialized allergy units, where sensitization is more common, and which cannot be generalized to the wider population.

Aligning with existing literature,^58^, ^59^ prevalence of sensitization to LTPs and storage proteins, which are commonly capable of causing (severe) systemic reactions,^60^ was generally higher in allergic individuals, and relatively rare in non‐allergic individuals. This was demonstrated in the study by Asarnoj et al,^38^ who found high sensitization prevalence to the major peanut storage protein allergens Ara h 1–3 among peanut‐allergic subjects, but not in those denying symptoms of allergy. Conversely, in non‐allergic subjects, sensitization was most common to Ara h 8, a cross‐reactive homologue to Bet v 1 from birch, belonging to the PR‐10 family,^48^, ^61^ a group of allergens reported to commonly cause mild, local reactions or no symptoms.^62^, ^63^, ^64^ Similarly, four out of the five most recognized allergens in the general population found in the study by Stemeseder et al.^42^ were PR‐10 proteins.

### 4.4 Implications for future research

Although molecular allergy diagnostics has increasingly been implemented over the last decade, this review found important research gaps in the field. In particular, only five studies published so far are population‐based, while most studies are case reports or case series, recruiting participants from hospitals or specialized inpatient/outpatient allergy clinics. These studies do not allow accurate estimation of population prevalence. Going forward, there is a need for more population‐based studies to accurately assess the prevalence of sensitization to molecular food allergens. Finally, given the current use of varying assays to measure IgE sensitization to molecular food allergens (e.g., microarray vs. single‐component tests), standardizing molecular allergy diagnostic assays will facilitate comparison between different tests.

## 5 CONCLUSION

This review, with data primarily from North‐western Europe, indicates that sensitization to molecular food allergens is overall low, particularly for molecular allergens representing primary sensitizers from the food, such as peanut storage proteins. The highest sensitization rates were seen for cross‐reactive PR‐10 proteins, for which birch pollen are acknowledged to be the primary sensitizer. More population‐representative studies are needed to gain a clearer appreciation of sensitization patterns to molecular food allergens in Europe.

## AUTHOR CONTRIBUTIONS

Bright I. Nwaru and Graham Roberts conceived the research question and developed the search strategy with assistance from Daniil Lisik. The data extraction form was developed by Daniil Lisik, with assistance from Bright I. Nwaru. Screening, data extraction, narrative synthesis, and writing of the manuscript was done by Daniil Lisik, Athina Ioannidou, and Bright I. Nwaru. Ekaterina Khaleva, Carina Venter, Ronald van Ree, Margitta Worm, Berber Vlieg‐Boerstra, Aziz Sheikh, Antonella Muraro, and Graham Roberts were consulted concerning methodology and synthesis of the findings. All authors critically commented on drafts of the manuscript.

## CONFLICT OF INTEREST

Carina Venter reports: grants (Reckitt Benckiser, Food Allergy Research and Education, and National Peanut Board) and personal fees (Reckitt Benckiser, Nestle Nutrition Institute, Danone, Abbott Nutrition, Else Nutrition, Sifter, and Before Brands). Ronald van Ree reports: consultancies (HAL Allergy BV, Citeq BV, Angany Inc., Reacta Healthcare Ltd., Mission MightyMe, and AB Enzymes), speaker's fees (HAL Allergy BV, ThermoFisher Scientific, and ALK), and stock options (Angany Inc.). Margitta Worm reports: grants and personal fees (Stallergens, HAL Allergie, Bencard Allergie, Allergopharma, ALK‐Abello, Mylan Germany, Actelion Pharmaceuticals Deutschland, Biotest, AbbVie Deutschland, Lilly Deutschland Aimmune, DBV Technologies SA, Regeneron Pharmaceuticals, Sanofi Aventis, Leo Pharma, Novartis, and Viatris) outside of the submitted work and being past WAO co‐chair of the anaphylaxis committee and past chair of the food allergy interest group of EAACI. Berber Vlieg‐Boerstra reports: personal fees (Marfo Food Group, Nestlé, and Nutricia) and grants (Nutricia). Antonella Muraro reports: grants and speaker's fees (Aimmune), speaker's fees (DVB Technologies SA, Viatris [Mylan], ALK, and Nestlé), and being member of the Executive Committee of GA2LEN and past president of EAACI. Graham Roberts reports grants (Asthma UK and National Institutes of Health Research). Bright Nwaru reports unrestricted grants and personal fees from DBV Technologies and AstraZeneca, respectively. The other authors report no conflicting interests related to this work. The funder played no role in the content and decision to submit this manuscript.

## Supporting information

Supporting Information S1

Supporting Information S2

Supporting Information S3

Supporting Information S4
