# Supplementary material for: Prevalence of sensitization to molecular food allergens in Europe: A systematic review
Source: Clin Transl Allergy. 2022 Jul 6;12(7):e12175. doi: 10.1002/clt2.12175 (PMC9260209; doi:10.1002/clt2.12175)
Supplement: Supplementary file 1 — Supporting Information S1 [file CLT2-12-e12175-s001.docx]

## Appendix 1: Geoscheme

| **Geoscheme of European countries as defined in the present study** | | | |
| --- | --- | --- | --- |
| **Eastern Europe** | **Northern Europe** | **Southern Europe** | **Western Europe** |
| Belarus | Åland* | Albania | Austria |
| Bulgaria | Channel Islands (Guernsey, Jersey, Sark) | Andorra | Belgium |
| Czech Republic | Denmark | Bosnia and Herzegovina | France |
| Hungary | Estonia | Croatia | Germany |
| Poland | Faroe Islands | Gibraltar | Liechtenstein |
| Moldova | Finland | Greece | Luxembourg |
| Romania | Iceland | Holy See (Vatican City) | Monaco |
| Russia | Ireland | Italy | Netherlands |
| Slovakia | Isle of Man | Kosovo* | Switzerland |
| Ukraine | Latvia | Malta |  |
|  | Lithuania | Montenegro |  |
|  | Norway | (North) Macedonia |  |
|  | Svalbard and Jan Mayen Islands* | Portugal |  |
|  | Sweden | San Marino |  |
|  | UK (England, Scotland, Wales, and Northern Ireland) | Serbia |  |
|  |  | Slovenia |  |
|  |  | Spain |  |
|  |  | Turkey* |  |
|  |  | Yugoslavia (historical)* |  |

Adapted version from <https://cies2018.org/wp-content/uploads/List-of-Countries-by-Region-UN-Annex-II.pdf> (accessed 6 April 2022).

* Appended to the original list due to subjective assessment of authors of geographical proximity to Europe.
