# Supplementary material for: Prevalence of sensitization to molecular food allergens in Europe: A systematic review
Source: Clin Transl Allergy. 2022 Jul 6;12(7):e12175. doi: 10.1002/clt2.12175 (PMC9260209; doi:10.1002/clt2.12175)
Supplement: Supplementary file 2 — Supporting Information S2 [file CLT2-12-e12175-s004.docx]

## Appendix 2: Search strategies

## Allergome

| **#** | **Search Terms (each done separately; see "Search strategy" in main paper for details)** |
| --- | --- |
| 1 | Almond |
| 2 | Brazil nut |
| 3 | Cashew |
| 4 | Egg |
| 5 | Hazelnut |
| 6 | Macadamia |
| 7 | Pecan |
| 8 | Pine nut |
| 9 | Pistachio |
| 10 | Shellfish |
| 11 | Fish |
| 12 | Milk |
| 13 | Peanut |
| 14 | Tree nut |
| 15 | Soy |
| 16 | Walnut |
| 17 | Wheat |

## Embase, MEDLINE, Scopus

| **#** | **Search terms** |
| --- | --- |
| 1 | exp Food Hypersensitivity/ or foodallerg*.mp. or food hypersensitivity.mp. or food hypersensitivities.mp. or food hypersensitivit*.mp. or food allerg*.mp. or exp hypersensitivity/ or exp food allergy/ or Peanut Hypersensitivity/ or peanut allergy.mp. or peanut allerg*.mp. or exp peanut allergy/ or Arachis hypogaea.mp. or exp Arachis/ or Ara h.mp. or Nuts/ or Nut Hypersensitivity/ or nut allergy.mp or nut allerg*.mp or nut hypersensitivit*.mp or tree nut allergy.mp or tree nut hypersensitivit*.mp or hazelnut allergy.mp or hazelnut hypersensitivit*.mp or cashew nut allergy.mp or cashew nut hypersensitivit*.mp or brazil nut allergy.mp or brazil nut hypersensitivit*.mp or almond allergy.mp or almond hypersensitivit*.mp. or Wheat Hypersensitivity/ or wheat allergy.mp. or wheat allerg*.mp. or wheat hypersensitivity*.mp. or exp wheat allergy/ or Soy Hypersensitivity/ or soy allergy.mp or soy allerg*.mp or soy hypersensitivit*.mp. or exp nut allergy/ or Egg Hypersensitivity/ or egg allergy.mp. or egg allerg*.mp. or egg hypersensitivity*.mp. or exp egg allergy/ or Milk Hypersensitivity/ or milk allergy.mp. or milk allerg*.mp. or cow milk allergy.mp. or cow milk allerg*.mp. or exp milk allergy/ or fish allergy.mp. or fish allerg*.mp. or fish hypersensitivit*.mp. or Shellfish Hypersensitivity/ or shellfish allergy.mp. or shellfish allerg*.mp. or shellfish hypersensitivit*.mp. or exp shellfish allergy/ or "sesame allergy".mp or seafood hypersensitivity.mp or sea food hypersensitivity.mp or Corylus avellana.mp. or Cor a.mp. or Anacardium occidentale.mp. or Ana o.mp. or Bertholletia excelsa.mp. or Ber e.mp. or Prunus dulcis.mp. or Pru du.mp. or Juglans regia.mp. or Jug r.mp. or Pistacia vera.mp. or Pis v.mp. or Glycine max.mp. or Gly m.mp. or Triticum aestivum.mp. or Tri a.mp. or Gallus domesticus.mp. or Gal d.mp. or Bos domesticus.mp. or Bos d.mp. or Penaeus aztecus.mp. or Pen a.mp. or Tropomyosin.mp. or exp Fabaceae/ or exp Peas/ or exp Cicer/ or exp Lens Plant/ or exp Vigna/ or exp Cajanus/ or (legume adj3 allerg*).mp or (legume adj3 hypersensitiv*).mp or (lentil adj3 allerg*).mp or (lentil adj3 hypersensitiv*).mp or (chickpea adj3 allerg*).mp or (chickpea adj3 hypersensitiv*).mp or (pea adj3 allerg*).mp or (pea adj3 hypersensitiv*).mp or ("mung bean" adj3 allerg*).mp or ("mung bean" adj3 hypersensitiv*).mp or ("red gram" adj3 allerg*).mp or ("red gram" adj3 hypersensitiv*) or ("pigeon pea" adj3 allerg*).mp or ("pigeon pea" adj3 hypersensitiv*).mp or exp Edible Insects/ or exp Hymenoptera/ or exp Insecta/ or "edible insect*".mp or hymenoptera.mp or "insect protein*".mp |
| 2 | exp Molecular Diagnostic Techniques/ or *Molecular Epidemiology/ or *Molecular Medicine/ or *Pathology, Molecular/ or exp Epitopes/ or molecular diagnos*.mp. or molecular diagnos*.ti,ab,su. or molecular allerg*.mp. or molecular allerg*.ti,ab,su. or immunoCap.mp. or immunoCap.ti,ab,su. or immuno-cap.mp or immuno-cap.su or component.mp. or component.ti,ab,su. or components.mp. or components.ti,ab,su. or allergen component*.mp. or allergen component*.ti,ab,su. or allergen components.mp. or allergen components.ti,ab,su. or molecular allergen test*.mp. or molecular allergen test*.ti,ab,su or allergen* molecule*.mp. or allergen* molecule*.ti,ab,su or component-resolved diagnos*.mp. or component-resolved diagnosis.ti,ab,su or component-resolved diagnostic.mp. or component-resolved diagnostic.ti,ab,su or component-resolved diagnostics.mp. or component-resolved diagnostics.ti,ab,su. |
| 3 | 1 and 2 |
| 4 | Incidence/ or incidence.mp or incidence.ti,ab,su or "incidence rate".mp or "incidence rate".ti,ab,su or rate.mp or rate.ti,ab,su or rates.mp or rates.ti,ab,su or "cumulative incidence".mp or "cumulative incidence".ti,ab,su or trend.mp or trend.ti,ab,su or trends.mp or trends.ti,ab,su or Prevalence/ or "prevalence rate".mp or "prevalence rates".mp or "prevalence rates".ti,ab,su or "prevalence rate".ti,ab,su or "point prevalence".mp or "point prevalence".ti,ab,su or "period prevalence".mp or "period prevalence".ti,ab,su or "life-time prevalence".mp or "life-time prevalence".ti,ab,su or "lifetime prevalence".mp or "lifetime prevalence".ti,ab,su or "prevalence trend".mp or "prevalence trend.ti,ab,su or "prevalence trends.mp or "prevalence trends".ti,ab,su or frequency.mp or proportion.mp or percentage.mp. or epidemiol*.mp or population.ti or review.mp or "meta-analysis".mp or survey.ti or exp population statistics/ or exp health statistics/ or exp "sample (statistics) "/ |
| 5 | 3 and 4 |
| 6 | exp Europe, Eastern/ or exp Europe/ or europe.mp or Austria-Hungary/ or Austria/ or Austria.mp or Belgium.mp or Belgium/ or Czech Republic.mp or exp Czechoslovakia/ or exp Czech Republic/ or Denmark.mp or exp Denmark/ or Estonia.mp or exp Estonia/ or Finland.mp or exp Finland/ or France.mp or exp France/ or exp Germany, East/ or germany.mp or exp Germany/ or exp Germany, West/ or Greece.mp or exp Greece/ or exp Hungary/ or Hungary.mp or Iceland.mp or exp Iceland/ or exp Northern Ireland/ or exp Ireland/ or Ireland.mp or Italy.mp or exp Italy/ or Luxembourg.mp or exp Luxembourg/ or exp Netherlands/ or Netherlands.mp or Norway.mp or exp Norway/ or exp Poland/ or Poland.mp or Portugal.mp or exp Portugal/ or Slovakia.mp or exp Slovakia/ or Slovenia.mp or exp Slovenia/ or Spain.mp or exp Spain/ or Sweden.mp or exp Sweden/ or Switzerland.mp or exp Switzerland/ or Turkey.mp or exp Turkey/ or United Kingdom.mp or exp United Kingdom/ or Great Britain.mp or exp Wales/ or Wales.mp or exp Scotland/ or Scotland.mp or exp England/ or England.mp or british isles.mp. or exp Scandinavia/ or Scandinavia.mp or exp Southern Europe/ or exp Western Europe/ or exp Baltic States/ or Baltic.mp. or Yugoslavia.mp or exp Yugoslavia/ or exp German Federal Republic/ or exp Latvia/ or Latvia.mp or exp Lithuania/ or Lithuania.mp |
| 7 | 5 and 6 |

## CINAHL

| **#** | **Search terms** |
| --- | --- |
| S1 | (MH "Food Hypersensitivity+") OR "food hypersensititiv*" OR "food allerg*" OR "allergy, food" OR "peanut hypersensitiv*" OR "peanut allerg*" OR "nut hypersensitiv*" OR "nut allerg*" OR "tree nut hypersensitiv*" OR "tree nut allerg*" OR "hazelnut hypersensitiv*" OR "hazelnut allerg*" OR "cashew nut hypersensitiv*" OR "cashew nut allerg*" OR "brazil nut hypersensitiv*" OR "brazil nut allerg*" OR "almond hypersensitiv*" OR "almond allerg*" OR "wheat hypersensitiv*" OR "wheat allerg'" OR "soy hypersensitiv*" OR "soy allerg*" OR "egg hypersensitiv*" OR "egg allerg*" OR (MH "Milk Hypersensitivity+") OR "milk hypersensitiv*" OR "milk allerg*" OR "cow's milk hypersensitiv*" OR "cow's milk allerg'" OR "cow milk hypersensitiv*" OR "cow milk allerg*" OR "fish hypersensitiv*" OR "fish allerg*" OR "shellfish hypersensitiv*" OR "shellfish allerg*" OR "sesame hypersensitiv*" OR "sesame allerg*" OR "seafood hypersensitiv*" OR "seafood allerg*" OR "sea food hypersensitiv*" OR "sea food allerg*" OR "Coryllus avellana" OR "Cor a" OR "Anacardium occidentale" OR "Ana o" OR "Bertholletia excelsa" OR "Ber e" OR "Prunus dulcis" OR "Pru du" OR "Juglans regia" OR "Jug r" OR "Pistacia vera" OR "Pis v" OR "Glycine max" OR "Gly m" OR "Triticum aestivum" OR "Tri a" OR "Gallus domesticus" OR "Gal d" OR "Bos domesticus" OR "Bos d" OR "Penaeus aztecus" OR "Pen a" OR "Tropomyosin" OR "Arachis hypogaea" OR "Ara h" OR (MH "Legumes+") OR "fabaceae" OR "legume*" OR legume N3 (allerg* OR hypersensitiv*) OR lentil N3 (allerg* OR hypersensitiv*) OR chickpea N3 (allerg* OR hypersensitiv*) OR pea N3 (allerg* OR hypersensitiv*) OR "mung bean" N3 (allerg* OR hypersensitiv*) OR "red gram" N3 (allerg* OR hypersensitiv*) OR "pigeon pea" N3 (allerg* OR hypersensitiv*) OR (MH "Insects+") OR "edible insect*" OR "hymenoptera" OR "insect protein*" |
| S2 | (MH "Molecular Diagnostic Techniques+") OR "molecular diagnostic techniques" OR (MH "Epidemiology, Molecular") OR "molecular epidemiology" OR (MH "Pathology, Molecular") OR (TI "epitope*") OR "molecular diagnos*" OR "molecular allerg*" OR "immunoCap" OR "immuno-cap" OR "immuno cap" OR "component*" OR "allergen component*" OR "molecular allergen test*" OR "allergen* molecule*" OR "component-resolved diagnos*" |
| S3 | (MH "Incidence+") OR "incidence" OR "incidence rate*" OR "rate*" OR "cumulative incidence" OR "trend*" OR (MH "Prevalence+") OR "prevalence*" OR "prevalence rate*" OR "point prevalence*" OR "period prevalence*" OR "life-time prevalence*" OR "lifetime prevalence*" OR "prevalence trend*" OR "frequency" OR "proportion*" OR "percentage*" OR "epidemiol*" OR (TI "population") OR (MH "Systematic Review+") OR (MH "Meta Analysis+") OR (TI "survey*") OR (MH "Population+") OR (MH "Urban Population+") OR (MH "Suburban Population+") OR (MH "Rural Population+") OR (MH "Population Characteristics+") OR (MH "Population Surveillance+") OR (MH "Population Health+") OR (MH "Demography+") |
| S4 | (MH "Europe+") OR (MH "Europe, Eastern+") OR "Europe" OR "Eastern Europe" OR "Western Europe" OR "Northern Europe" OR "Southern Europe" OR "Austria-Hungary" OR (MH "Austria") OR (MH "Belgium") OR "Belgium" OR (MH "Czech Republic") OR (MH "Czechoslovakia") OR "Czech*" OR (MH "Denmark") OR "Denmark" OR (MH "Estonia") OR "Estonia" OR (MH "Finland") OR "Finland" OR (MH "France") OR "France" OR (MH "Germany") OR (MH "Germany, East") OR (MH "Germany, West") OR "Germany" OR (MH "Greece") OR "Greece" OR (MH "Hungary") OR "Hungary" OR (MH "Iceland") OR "Iceland" OR (MH "Northern Ireland") OR "Ireland" OR (MH "Ireland") OR (MH "Italy") OR "Italy" OR (MH "Luxembourg") OR "Luxembourg" OR (MH "Netherlands") OR "Netherlands" OR (MH "Norway") OR "Norway" OR (MH "Poland") OR "Poland" OR (MH "Portugal") OR "Portugal" OR (MH "Slovakia") OR "Slovakia" OR (MH "Slovenia") OR "Slovenia" OR (MH "Spain") OR "Spain" OR (MH "Sweden") OR "Sweden" OR (MH "Switzerland") OR "Switzerland" OR (MH "Turkey") OR "Turkey" OR (MH "United Kingdom") OR "United Kingdom" OR "Great Britain" OR (MH "Great Britain") OR (MH "Scotland") OR (MH "England") OR (MH "Wales") OR "British Isles" OR "Scotland" OR "England" OR "Wales" OR (MH "Scandinavia") OR "Scandinavia" OR (MH "Baltic States") OR "Baltic" OR (MH "Yugoslavia") OR "German Federal Republic" OR (MH "Latvia") OR "Latvia" OR (MH "Lithuania") OR "Lithuania" |
| S5 | S1 AND S2 |
| S6 | S3 AND S4 |
| S7 | S5 AND S6 |

## Cochrane Library

| **#** | **Search terms** |
| --- | --- |
| 1 | MeSH descriptor: [Food Hypersensitivity] explode all trees |
| 2 | MeSH descriptor: [Hypersensitivity] explode all trees |
| 3 | MeSH descriptor: [Peanut Hypersensitivity] explode all trees |
| 4 | MeSH descriptor: [Arachis] explode all trees |
| 5 | MeSH descriptor: [Nuts] explode all trees |
| 6 | MeSH descriptor: [Nut Hypersensitivity] explode all trees |
| 7 | MeSH descriptor: [Wheat Hypersensitivity] explode all trees |
| 8 | MeSH descriptor: [Egg Hypersensitivity] explode all trees |
| 9 | MeSH descriptor: [Milk Hypersensitivity] explode all trees |
| 10 | MeSH descriptor: [Shellfish Hypersensitivity] explode all trees |
| 11 | MeSH descriptor: [Corylus] explode all trees |
| 12 | MeSH descriptor: [Anacardium] explode all trees |
| 13 | MeSH descriptor: [Bertholletia] explode all trees |
| 14 | MeSH descriptor: [Prunus dulcis] explode all trees |
| 15 | MeSH descriptor: [Juglans] explode all trees |
| 16 | MeSH descriptor: [Pistacia] explode all trees |
| 17 | MeSH descriptor: [Soybeans] explode all trees |
| 18 | MeSH descriptor: [Triticum] explode all trees |
| 19 | MeSH descriptor: [Chickens] explode all trees |
| 20 | MeSH descriptor: [Penaeidae] explode all trees |
| 21 | MeSH descriptor: [Tropomyosin] explode all trees |
| 22 | (foodallerg*):ti,ab,kw OR (food-allerg*):ti,ab,kw OR (food allerg*):ti,ab,kw OR (food hypersensitiv*):ti,ab,kw OR (hypersensitiv*):ti,ab,kw |
| 23 | MeSH descriptor: [Fabaceae] explode all trees |
| 24 | MeSH descriptor: [Peas] explode all trees |
| 25 | MeSH descriptor: [Cicer] explode all trees |
| 26 | MeSH descriptor: [Lens Plant] explode all trees |
| 27 | MeSH descriptor: [Vigna] explode all trees |
| 28 | MeSH descriptor: [Cajanus] explode all trees |
| 29 | MeSH descriptor: [Edible Insects] explode all trees |
| 30 | MeSH descriptor: [Hymenoptera] explode all trees |
| 31 | MeSH descriptor: [Insecta] explode all trees |
| 32 | (legume NEAR/3 allerg*):ti,ab,kw OR (legume NEAR/3 hypersensitiv*):ti,ab,kw OR (lentil NEAR/3 allerg*):ti,ab,kw OR (lentil NEAR/3 hypersensitiv*):ti,ab,kw OR (chickpea NEAR/3 allerg*):ti,ab,kw |
| 33 | (chickpea NEAR/3 hypersensitiv*):ti,ab,kw OR (pea NEAR/3 allerg*):ti,ab,kw OR (pea NEAR/3 hypersensitiv*):ti,ab,kw OR ("mung bean" NEAR/3 allerg*):ti,ab,kw OR ("mung bean" NEAR/3 hypersensitiv*):ti,ab,kw |
| 34 | ("red gram" NEAR/3 allerg*):ti,ab,kw OR ("red gram" NEAR/3 hypersensitiv*):ti,ab,kw OR ("pigeon pea" NEAR/3 allerg*):ti,ab,kw OR ("pigeon pea" NEAR/3 hypersensitiv*):ti,ab,kw OR (edible insect*):ti,ab,kw |
| 35 | (hymenoptera):ti,ab,kw OR (insect protein*):ti,ab,kw |
| 36 | (peanut allerg*):ti,ab,kw OR (peanut hypersensitiv*):ti,ab,kw OR (nut allerg*):ti,ab,kw OR (nut hypersensitiv*):ti,ab,kw OR (tree nut allerg*):ti,ab,kw |
| 37 | (tree nut hypersensitiv*):ti,ab,kw OR (hazelnut allerg*):ti,ab,kw OR (hazelnut hypersensitiv*):ti,ab,kw OR (cashew nut allerg*):ti,ab,kw OR (cashew nut hypersensitiv*):ti,ab,kw |
| 38 | (brazil nut allerg*):ti,ab,kw OR (brazil nut hypersensitiv*):ti,ab,kw OR (almond allerg*):ti,ab,kw OR (almond hypersensitiv*):ti,ab,kw OR (wheat allerg*):ti,ab,kw |
| 39 | (wheat hypersensitiv*):ti,ab,kw OR (soy allerg*):ti,ab,kw OR (soy hypersensitiv*):ti,ab,kw OR (egg allerg*):ti,ab,kw OR (egg hypersensitiv*):ti,ab,kw |
| 40 | (milk allerg*):ti,ab,kw OR (milk hypersensitiv*):ti,ab,kw OR (cow milk allerg*):ti,ab,kw OR (cow milk hypersensitiv*):ti,ab,kw OR (cow's milk allerg*):ti,ab,kw |
| 41 | (cow's milk hypersensitiv*):ti,ab,kw OR (fish allerg*):ti,ab,kw OR (fish hypersensitiv*):ti,ab,kw OR (shellfish allerg*):ti,ab,kw OR (shellfish hypersensitiv*):ti,ab,kw |
| 42 | (sesame allerg*):ti,ab,kw OR (sesame hypersensitiv*):ti,ab,kw OR (seafood allerg*):ti,ab,kw OR (seafood hypersensitiv*):ti,ab,kw OR (sea food allerg*):ti,ab,kw |
| 43 | (sea food hypersensitiv*):ti,ab,kw OR (Arachis hypogaea):ti,ab,kw OR (Ara h):ti,ab,kw OR (Corylus avellana):ti,ab,kw OR (Cor a):ti,ab,kw |
| 44 | (Anacardium occidentale):ti,ab,kw OR (Ana o):ti,ab,kw OR (Bertholletia excelsa):ti,ab,kw OR (Ber e):ti,ab,kw OR (Prunus dulcis):ti,ab,kw |
| 45 | (Pru du):ti,ab,kw OR (Juglans regia):ti,ab,kw OR (Jug r):ti,ab,kw OR (Pistacia vera):ti,ab,kw OR (Pis v):ti,ab,kw |
| 46 | (Glycine max):ti,ab,kw OR (Gly m):ti,ab,kw OR (Triticum aestivum):ti,ab,kw OR (Tri a):ti,ab,kw OR (Gallus domesticus):ti,ab,kw |
| 47 | (Gal d):ti,ab,kw OR (Bos domesticus):ti,ab,kw OR (Bos d):ti,ab,kw OR (Penaeus aztecus):ti,ab,kw OR (Pen a):ti,ab,kw |
| 48 | (Tropomyosin):ti,ab,kw |
| 49 | {OR #1-#48} |
| 50 | MeSH descriptor: [Molecular Diagnostic Techniques] explode all trees |
| 51 | MeSH descriptor: [Molecular Epidemiology] explode all trees |
| 52 | MeSH descriptor: [Molecular Medicine] explode all trees |
| 53 | MeSH descriptor: [Pathology, Molecular] explode all trees |
| 54 | MeSH descriptor: [Epitopes] explode all trees |
| 55 | (molecular diagnos*):ti,ab,kw OR (molecular allerg*):ti,ab,kw OR (ImmunoCap):ti,ab,kw OR (immuno-Cap):ti,ab,kw OR ("immuno cap"):ti,ab,kw |
| 56 | (component*):ti,ab,kw OR (allergen component*):ti,ab,kw OR (molecular allergen test*):ti,ab,kw OR (allergen* molecule*):ti,ab,kw OR (component-resolved diagnos*):ti,ab,kw |
| 57 | {OR #50-#56} |
| 58 | MeSH descriptor: [Incidence] explode all trees |
| 59 | MeSH descriptor: [Prevalence] explode all trees |
| 60 | MeSH descriptor: [Cross-Sectional Studies] explode all trees |
| 61 | MeSH descriptor: [Population Characteristics] explode all trees |
| 62 | MeSH descriptor: [Epidemiology] explode all trees |
| 63 | (survey*):ti OR (population*):ti |
| 64 | MeSH descriptor: [Systematic Review] explode all trees |
| 65 | (epidemiol*):ti,ab,kw OR (review):ti,ab,kw OR (meta-analysis):ti,ab,kw |
| 66 | (incidence*):ti,ab,kw OR (incidence rate*):ti,ab,kw OR (rate*):ti,ab,kw OR (cumulative incidence*):ti,ab,kw OR (trend*):ti,ab,kw |
| 67 | (prevalence rate*):ti,ab,kw OR (point prevalence*):ti,ab,kw OR (period prevalence*):ti,ab,kw OR (life-time prevalence*):ti,ab,kw OR (lifetime prevalence*):ti,ab,kw |
| 68 | (prevalence trend*):ti,ab,kw OR (frequenc*):ti,ab,kw OR (proportion*):ti,ab,kw OR (percentage*):ti,ab,kw |
| 69 | {OR #58-#68} |
| 70 | MeSH descriptor: [Europe] explode all trees |
| 71 | MeSH descriptor: [Europe, Eastern] explode all trees |
| 72 | (Western Europe):ti,ab,kw AND (Northern Europe):ti,ab,kw AND (Southern Europe):ti,ab,kw AND (Eastern Europe):ti,ab,kw |
| 73 | MeSH descriptor: [Austria-Hungary] explode all trees |
| 74 | MeSH descriptor: [Austria] explode all trees |
| 75 | MeSH descriptor: [Belgium] explode all trees |
| 76 | MeSH descriptor: [Czech Republic] explode all trees |
| 77 | MeSH descriptor: [Czechoslovakia] explode all trees |
| 78 | MeSH descriptor: [Denmark] explode all trees |
| 79 | MeSH descriptor: [Estonia] explode all trees |
| 80 | MeSH descriptor: [Finland] explode all trees |
| 81 | MeSH descriptor: [France] explode all trees |
| 82 | MeSH descriptor: [Germany] explode all trees |
| 83 | MeSH descriptor: [Germany, East] explode all trees |
| 84 | MeSH descriptor: [Germany, West] explode all trees |
| 85 | MeSH descriptor: [Greece] explode all trees |
| 86 | MeSH descriptor: [Hungary] explode all trees |
| 87 | MeSH descriptor: [Iceland] explode all trees |
| 88 | MeSH descriptor: [Northern Ireland] explode all trees |
| 89 | MeSH descriptor: [Ireland] explode all trees |
| 90 | MeSH descriptor: [Italy] explode all trees |
| 91 | MeSH descriptor: [Luxembourg] explode all trees |
| 92 | MeSH descriptor: [Netherlands] explode all trees |
| 93 | MeSH descriptor: [Norway] explode all trees |
| 94 | MeSH descriptor: [Poland] explode all trees |
| 95 | MeSH descriptor: [Portugal] explode all trees |
| 96 | MeSH descriptor: [Slovakia] explode all trees |
| 97 | MeSH descriptor: [Slovenia] explode all trees |
| 98 | MeSH descriptor: [Spain] explode all trees |
| 99 | MeSH descriptor: [Sweden] explode all trees |
| 100 | MeSH descriptor: [Switzerland] explode all trees |
| 101 | MeSH descriptor: [United Kingdom] explode all trees |
| 102 | MeSH descriptor: [Wales] explode all trees |
| 103 | MeSH descriptor: [Scotland] explode all trees |
| 104 | MeSH descriptor: [England] explode all trees |
| 105 | MeSH descriptor: [Scandinavian and Nordic Countries] explode all trees |
| 106 | MeSH descriptor: [Baltic States] explode all trees |
| 107 | MeSH descriptor: [Yugoslavia] explode all trees |
| 108 | MeSH descriptor: [Latvia] explode all trees |
| 109 | MeSH descriptor: [Lithuania] explode all trees |
| 110 | (Europe):ti,ab,kw OR (Eastern Europe):ti,ab,kw OR (Western Europe):ti,ab,kw OR (Northern Europe):ti,ab,kw OR (Southern Europe):ti,ab,kw |
| 111 | (Austria-Hungary):ti,ab,kw AND (Austria):ti,ab,kw AND (Belgium):ti,ab,kw AND (Czech*):ti,ab,kw AND (Denmark):ti,ab,kw |
| 112 | (Estonia):ti,ab,kw OR (Finland):ti,ab,kw OR (France):ti,ab,kw OR (Germany):ti,ab,kw OR (Greece):ti,ab,kw |
| 113 | (Hungary):ti,ab,kw OR (Iceland):ti,ab,kw OR (Ireland):ti,ab,kw OR (Italy):ti,ab,kw OR (Luxembourg):ti,ab,kw |
| 114 | (Netherlands):ti,ab,kw OR (Norway):ti,ab,kw OR (Poland):ti,ab,kw OR (Portugal):ti,ab,kw OR (Slovakia):ti,ab,kw |
| 115 | (Slovenia):ti,ab,kw OR (Spain):ti,ab,kw OR (Sweden):ti,ab,kw OR (Switzerland):ti,ab,kw OR (Turkey):ti,ab,kw |
| 116 | (United Kingdom):ti,ab,kw OR (Great Britain):ti,ab,kw OR (Wales):ti,ab,kw OR (England):ti,ab,kw OR (Scotland):ti,ab,kw |
| 117 | (British Isles):ti,ab,kw OR (Scandinavia):ti,ab,kw OR (Nordic countr*):ti,ab,kw OR (Baltic State*):ti,ab,kw OR (Yugoslavia):ti,ab,kw |
| 118 | (German):ti,ab,kw OR (Latvia):ti,ab,kw OR (Lithuania):ti,ab,kw |
| 119 | [#70-#118] |
| 120 | #49 AND #57 AND #69 AND #119 |

## Web of Science

| **#** | **Search terms** |
| --- | --- |
| #1 | TS="hypersensitiv*" OR TS="food hypersensitiv*" OR TS="food allerg*" OR TS="food-allerg*" OR TS="foodallerg*" OR TS="allergy, food" OR TS="peanut hypersensitiv*" OR TS="peanut allerg*" OR TS="nut hypersensitiv*" OR TS="nut allerg*" OR TS="tree nut hypersensitiv*" OR TS="tree nut allerg*" OR TS="hazelnut hypersensitiv*" OR TS="hazelnut allerg*" OR TS="cashew nut hypersensitiv*" OR TS="cashew nut allerg*" OR TS="cashewnut hypersensitiv*" OR TS="cashewnut allerg*" OR TS="brazil nut hypersensitiv*" OR TS="brazil nut allerg*" OR TS="almond nut hypersensitiv*" OR TS="almond nut allerg*" OR TS="almond hypersensitiv*" OR TS="almond allerg*" OR TS="wheat hypersensitiv*" OR TS="wheat allerg*" OR TS="soy hypersensitiv*" OR TS="soy allerg*" OR TS="soybean hypersensitiv*" OR TS="soybean allerg*" OR TS="egg hypersensitiv*" OR TS="egg allerg*" OR TS="milk hypersensitiv*" OR TS="milk allerg*" OR TS="cow milk's hypersensitiv*" OR TS="cow milk's allerg*" OR TS="fish hypersensitiv*" OR TS="fish allerg*" OR TS="shellfish hypersensitiv*" OR TS="shellfish allerg*" OR TS="sesame hypersensitiv*" OR TS="sesame allerg*" OR TS="seafood hypersensitiv*" OR TS="seafood allerg*" OR TS="sea food hypersensitiv*" OR TS="sea food allerg*" OR TS="corylus avellana" OR TS="cor a" OR TS="anacardium occidentale" OR TS="ana o" OR TS="bertholletia excelsa" OR TS="ber e" OR TS="prunus dulcis" OR TS="pru du" OR TS="juglans regia" OR TS="jug r" OR TS="pistacia vera" OR TS="pis v" OR TS="glycine max" OR TS="gly m" OR TS="triticum aestivum" OR TS="tri a" OR TS="gallus domesticus" OR TS="gal d" OR TS="bos domesticus" OR TS="bos d" OR TS="penaeus aztecus" OR TS="pen a" OR TS="troptomyosin" OR TS="Arachis hypogaea" OR TS="Ara h" OR TS="fabaceae" OR TS="Cicer" OR TS="Vigna" OR TS="Cajanus" OR TS="legume*" OR TS=(lentil NEAR/3 (allerg* OR hypersensitiv*)) OR TS=(chickpea NEAR/3 (allerg* OR hypersensitiv*)) OR TS=(pea NEAR/3 (allerg* OR hypersensitiv*)) OR TS=("mung bean" NEAR/3 (allerg* OR hypersensitiv*)) OR TS=("red gram" NEAR/3 (allerg* OR hypersensitiv*)) OR TS=("pigeon pea" NEAR/3 (allerg* OR hypersensitiv*)) OR TS="edible insect*" OR TS="hymenoptera" OR TS="insect protein*" |
| #2 | TS="molecular diagnostic techniques" OR TS="molecular epidemiology" OR TS="molecular medicine" OR TS="molecular pathology" OR TS="pathology, molecular" OR TS="epitopes" OR TS="molecular diagnos*" OR TS="molecular allerg*" OR TS="immunocap" OR TS="immuno cap" OR TS="immuno-cap" OR TS="component*" OR TS="allergen component*" OR TS="molecular allergen test*" OR TS="allergen* molecule*" OR TS="component-resolved diagnos*" |
| #3 | TS="incidence*" OR TS="incidence rate*" OR TS="rate*" OR TS="cumulative incidence*" OR TS="prevalence*" OR TS="prevalence rate*" OR TS="point prevalence*" OR TS="period prevalence*" OR TS="life-time prevalence*" OR TS="lifetime prevalence*" OR TS="lifetime trend*" OR TS="prevalence trend*" OR TS="incidence trend*" OR TS="frequency" OR TS="proportion*" OR TS="percentage*" OR TS="epidemiol*" OR TI="population*" OR TS="REVIEW" OR TS="META-ANALYSIS" OR TI="SURVEY*" OR TS="POPULATION STATISTIC*" OR TS="HEALTH STATISTIC*" |
| #4 | TS="Europe, Eastern" OR TS="Europe" OR TS="Austria-Hungary" OR TS="Austria" OR TS="Belgium" OR TS="Czech Republic" OR TS="Czechoslovakia" OR TS="Denmark" OR TS="Estonia" OR TS="Finland" OR TS="France" OR TS="Germany" OR TS="Germany, East" OR TS="Germany, West" OR TS="Greece" OR TS="Hungary" OR TS="Iceland" OR TS="Ireland" OR TS="Northern Ireland" OR TS="Ireland" OR TS="Italy" OR TS="Luxembourg" OR TS="Netherlands" OR TS="Norway" OR TS="Poland" OR TS="Portugal" OR TS="Slovakia" OR TS="Slovenia" OR TS="Spain" OR TS="Sweden" OR TS="Switzerland" OR TS="Turkey" OR TS="United Kingdom" OR TS="Great Britain" OR TS="Wales" OR TS="Scotland" OR TS="England" OR TS="British Isles" OR TS="Scandinavia" OR TS="Southern Europe" OR TS="Eastern Europe" OR TS="Europe, Southern" OR TS="Europe, Eastern" OR TS="Western Europe" OR TS="Europe, Western" OR TS="Northern Europe" OR TS="Europe, Northern" OR TS="Baltic States" OR TS="Baltic" OR TS="Yugoslavia" OR TS="German Federal Republic" OR TS="Latvia" OR TS="Lithuania" |
| #5 | #1 AND #2 |
| #6 | #3 AND #4 |
| #7 | #5 AND #6 |
