# Supplementary material for: Prevalence of sensitization to molecular food allergens in Europe: A systematic review
Source: Clin Transl Allergy. 2022 Jul 6;12(7):e12175. doi: 10.1002/clt2.12175 (PMC9260209; doi:10.1002/clt2.12175)
Supplement: Supplementary file 4 — Supporting Information S4 [file CLT2-12-e12175-s003.docx]

## Appendix 4: Disagreements in second stage of screening

| **Study reference** | **Disagreement** | **Arbitration required** | **Final decision** |
| --- | --- | --- | --- |
| Alessandri, C., Guggiari Doutreleau, J., Mardones, P., Pietrosanti, I., Ciancamerla, M., Rafaiani, C., Ciardiello, M. A., & Mari, A. (2018). A standard diagnostic tool to explore allergic sensitization across distant countries in two continents [Conference Abstract]. Allergy: European Journal of Allergy and Clinical Immunology, 73, 366-367. https://doi.org/10.1111/all.13538 | Whether the study sample is population-representative | No | Exclude |
| D'Amelio, C. M., Ferrer, M. M., Martínez-Aranguren, R. M., Martín-Cuesta, J., Goikoetxea, M. J., & Gastaminza, G. (2018). DIAGNOSTIC PERFORMANCE OF DIFFERENT METHODS FOR DETECTION OF SENSITIZATION TO PRU P 3 IN SPAIN. Journal of Allergy and Clinical Immunology, 141(2), AB152. <https://doi.org/10.1016/j.jaci.2017.12.485> | Usability of data for the present review | No | Exclude |
| Eller, E., & Bindslev-Jensen, C. (2012). The course of clinical thresholds and sensitisation in peanut allergic patients [Conference Abstract]. Allergy: European Journal of Allergy and Clinical Immunology, 67, 87. <https://doi.org/10.1111/all.12033> | Usability of data for the present review | No | Exclude |
| Fernandez, F. J., Flores-Pardo, E., Moreno, M. V., Caparros, E., Velasquez, L. I., & Gómez, F. (2016). Clinical and Immunological Profile of Patients Allergic to Fruits in East of Spain. Journal of Allergy and Clinical Immunology, 137(2), AB155. <https://doi.org/10.1016/j.jaci.2015.12.634> | Whether the study sample is population-representative | No | Exclude |
| Griffiths, R., El-Shanawany, T., Jolles, S., Selwood, C., Heaps, A. G., Carne, E. M., & Williams, P. E. (2017). Comparison of the Performance of Skin Prick, ImmunoCAP, and ISAC Tests in the Diagnosis of Patients with Allergy. *International archives of allergy and immunology*, *172*(4), 215–223. <https://doi.org/10.1159/000464326> | Usability of data for the present review | No | Excldue |
| Uotila, R. T. I., Kukkonen, A. K., Pelkonen, A. S., & Mäkelä, M. J. (2016). Specific IgG4/IgE ratios to Ara h 1, 2, 3 and whole peanut extract serve as markers for clinical reactivity to peanut [Conference Abstract]. Allergy: European Journal of Allergy and Clinical Immunology, 71, 134. <https://doi.org/10.1111/all.12972> | Usability of data for the present review | No | Exclude |
| Lee, J., Jeon, S., & Lee, S. (2012). Analysis of cow's milk-specific IgE and components IgE antibodies in infants and young children with atopic dermatitis [Conference Abstract]. Allergy: European Journal of Allergy and Clinical Immunology, 67, 119-120. <https://doi.org/10.1111/all.12034> | Whether the study takes place in Europe | No | Excude |
| Fedorova, O. S., Ogorodova, L. M., Fedotova, M. M., Evdokimova, T. A., Deev, I. A., Deeva, E. V., Kamaltynova, E. M., Kolomeets, I. L., Kulikov, P. A., Selivanova, P. A., Spitsko Zh, A., & Starovoĭtova, E. A. (2013). [The prevalence of food allergies in children in world nidus of opisthorchiasis: background and study methodology of epidemiological survey EuroPrevall]. Vestn Ross Akad Med Nauk(4), 18-24. <https://doi.org/10.15690/vramn.v68i4.606> | Whether the study design is correct for the present review | No | Exclude |
| Majsiak, E., Kurzawa, R., Choina, M., & Miskiewicz, K. (2019). The first comparison of two multiparameter methods to measure allergen specific IgE among the polish group of kids suffering from allergy [Conference Abstract]. Allergy: European Journal of Allergy and Clinical Immunology, 74, 628. <https://doi.org/10.1111/all.13961> | Whether the study fulfills inclusion criteria (measuring prevalence of sensitization to molecular allergens) | No | Exclude |
| Eriksson, C., Kekki, O. M., Brostedt, P., Sjolander, S., & Turjanmaa, K. (2013). Wheat gliadins improve the specificity of IgE antibody testing in a group of wheat allergic Finnish children [Conference Abstract]. Allergy: European Journal of Allergy and Clinical Immunology, 68, 129. <https://doi.org/10.1111/all.12249> | Whether the study sample is population-representative | No | Exclude |
| Santos, M. C. P., Silva, P., Costa, C., Pestana, L., & Barbosa, M. P. (2014). Frequency of LTP (Pru p 3) and Profilin (Pru p 4) sensitisation in 1052 patients referenced to an immunoallergology department in Lisbon [Conference Abstract]. Clinical and Translational Allergy, 4. <https://www.embase.com/search/results?subaction=viewrecord&id=L71703183&from=export> | Whether the study sample is population-representative | No | Exclude |
| Scala, E., Abeni, D., Cecchi, L., Guerra, E. C., Locanto, M., Pirrotta, L., Giani, M., & Asero, R. (2017). Molecular Recognition Profiles and Clinical Patterns of PR-10 Sensitization in a Birch-Free Mediterranean Area. *International archives of allergy and immunology*, *173*(3), 138–146. <https://doi.org/10.1159/000477565> | Whether the study sample is population-representative | No | Exclude |
| Scala, E., Alessandri, C., Palazzo, P., Pomponi, D., Liso, M., Bernardi, M., Ferrara, R., Zennaro, D., Santoro, M., Rasi, C., & Mari, A. (2011). Allergenic molecule-based microarraybased allergy diagnosis: The second “Real life” Study on a cohort of 23,559 subjects [Conference Abstract]. Allergy: European Journal of Allergy and Clinical Immunology, 66, 100. <https://doi.org/10.1111/j.1398-9995.2011.02604.x> | Whether the study sample is population-representative | No | Exclude |
| Majsiak, E., Choina, M., Miskiewicz, K., & Kurzawa, R. (2020). Verification of new diagnostic method among polish patients with allergy [Conference Abstract]. Allergy: European Journal of Allergy and Clinical Immunology, 75(SUPPL 109), 579. <https://doi.org/10.1111/all.14509> | Whether the study fulfills inclusion criteria (measuring prevalence of sensitization to molecular allergens) | No | Exclude |
| Ackerbauer, D., Bublin, M., Radauer, C., Varga, E. M., Hafner, C., Ebner, C., Szépfalusi, Z., Fröschl, R., Hoffmann-Sommergruber, K., Eiwegger, T., & Breiteneder, H. (2015). Component-resolved IgE profiles in Austrian patients with a convincing history of peanut allergy. *International archives of allergy and immunology*, *166*(1), 13–24. <https://doi.org/10.1159/000371422> | Whether the study sample is population-representative | No | Exclude |
| Agabriel, C., Ghazouani, O., Birnbaum, J., Liabeuf, V., Porri, F., Gouitaa, M., Cleach, I., Grob, J. J., Bongrand, P., Sarles, J., & Vitte, J. (2014). Ara h 2 and Ara h 6 sensitization predicts peanut allergy in Mediterranean pediatric patients. *Pediatric allergy and immunology : official publication of the European Society of Pediatric Allergy and Immunology*, *25*(7), 662–667. <https://doi.org/10.1111/pai.12299> | Whether the study sample is population-representative | No | Exclude |
| Balková, E. (2015). Oral allergy syndrome and pollen food allergy syndrome. Lekarsky Obzor, 64(6), 207-211. <https://www.scopus.com/inward/record.uri?eid=2-s2.0-85020164751&partnerID=40&md5=3428d8fd931db2af1547a87935422a7d> | Usability of data for the present review | No | Exclude |
| Belohlávková, S., Kopelentová, E., Víšek, P., Šetinová, I., & Galanská, R. (2019). Epidemiology of food allergy in Czech Republic, final results of dafall registry [Conference Abstract]. Allergy: European Journal of Allergy and Clinical Immunology, 74, 511-512. <https://doi.org/10.1111/all.13961> | Whether the study fulfills inclusion criteria (measuring prevalence of sensitization to molecular allergens) | No | Exclude |
| Bernardi, M. L., Giangrieco, I., Camardella, L., Panico, M. R., Zennaro, D., Ferrara, R., Palazzo, P., Tuppo, L., Tamburrini, M., Carratore, V., Santoro, M., Ciardiello, M. A., & Mari, A. (2011). Biochemical and immunological characterisation of Act d 10 a lipid transfer proteins from green kiwifruit [Conference Abstract]. Clinical and Translational Allergy, 1. <https://www.embase.com/search/results?subaction=viewrecord&id=L71388042&from=export> | Whether the study fulfills inclusion criteria (measuring prevalence of sensitization to molecular allergens) | No | Exclude |
